# Carbonization of a radicular cyst using fiber-optic diode laser: a case report

**DOI:** 10.1186/1757-1626-1-113

**Published:** 2008-08-19

**Authors:** Panagiotis Kafas, Sotirios Kalfas

**Affiliations:** 1 Department of Oral Surgery, School of Dentistry, Aristotle University, Thessalonica, Greece; 2 Department of Preventive Dentistry, School of Dentistry, Aristotle University, Thessalonica, Greece

## Abstract

A female patient, 51 years old, complaint of painful swelling on the anatomical area of the upper left lateral incisor. The diagnosis of radicular cyst was confirmed histo-pathologically. Nowadays, radicular cysts may be treated using conventional root canal methods or surgical apicectomy. The possible soft-laser reaction to radicular cysts after contact application has not been investigated. We present an in vitro case of a diagnosed radicular cyst which carbonized after contact application of diode laser. The need for future clinical trials will be essential to prove the sensitivity of this procedure in humans.

## Case report

A Caucasian female patient, 51 years old presented to the clinic with swelling of the anterior upper jaw. The patient was working is store house. She was smoking 20 cigarettes per day for 20 years. She was drinking alcohol occasionally. The medical history revealed previous surgical procedure for appendicectomy. The dental history was free apart from routine dental procedures such as fillings and prosthetics. The patient was 58 kg weight and 167 cm height. The clinical and radiographic examination revealed a periapical lesion of the upper left lateral incisor.

It was decided to extract the tooth which was severely decayed and non restorable. The upper left incisor was extracted with the associated periapical lesion. The tooth with the soft-tissue lesion was stored in buffered formalin for 24 hours and decalcified. The decalcification was carried out to cut the tooth longitudinally. This allowed the processing of specimens for their diagnostic microscopic evaluation. The pathological examination showed the atrophic epithelial lining of radicular cyst.

**Figure 1:**
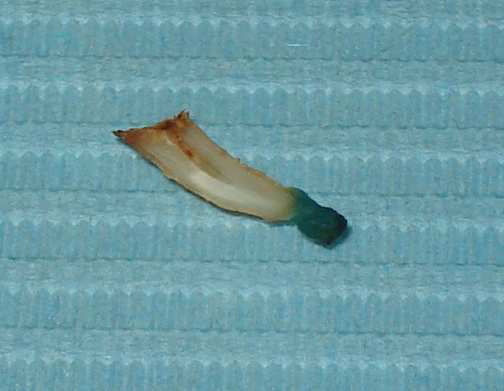
Radicular cyst size was observed using methylene blue staining.

**Figure 2:**
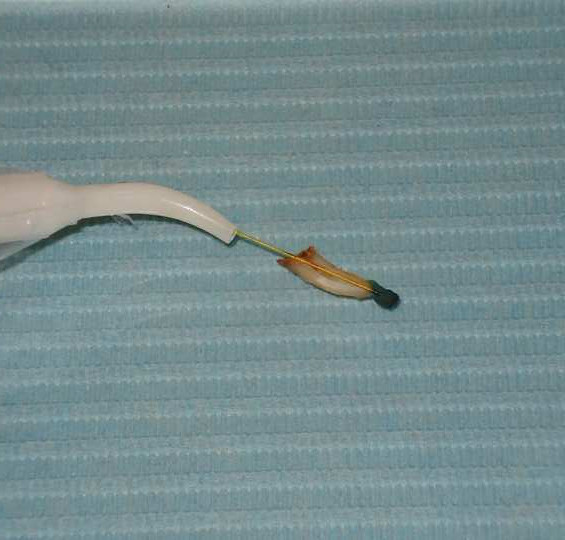
Fiber-optic passed through the bisected root canal into the cystic cavity.

The second tooth and cyst portion was used for evaluation of laser performance. The laser parameters used were 1500 mW of continuous output in 808 nm wavelength. The fiber optic had a diameter of 300 μm. The cyst was stained using methylene blue for observation (Figure. 1). The fiber-optic diode laser passed through the bisected root canal into the radicular cyst (Figure. 2). The cyst carbonized in about 30 seconds after constant contact. It was obvious that the destruction of radicular cysts may be carried out with laser techniques. Theoretically, this will allow rapid healing if it is associated with endodontic treatment.

## Discussion

Radicular cysts are the most common inflammatory tooth-associated cystic lesions [1]. Their treatment is mainly based on lege artis endodontic treatment or surgical excision of the cyst lining with apicectomy [2,3]. These two well established procedures are considered the golden standards for the management of such lesions.

A diode laser causes warming, welding, coagulation, protein denaturization, drying, vaporization, and carbonization of the target tissue [4]. Diode-laser treatment of targeted periapical lesions has not been used in the past.

**Figure 3:**
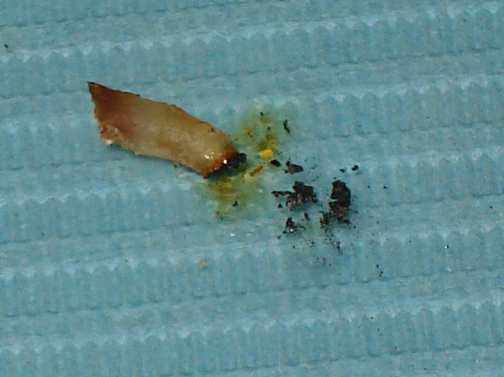
Carbonization of radicular cyst was performed in a few seconds.

The reason for the skepticism about this procedure may be associated to the complete digestion of carbonized products (Figure. 3). According to previous reports, carbonized tissues are digested by giant cells or macrophages [5].

Diode-laser carbonization of radicular cysts may be effective using fiber-optic technology. The only prerequisite is the measurement of the tooth length for correctly placing the fiber through the root canal into the cystic cavity. Hypothetically, the limitations to the application of this technique maybe the impaired defense mechanism, the smoke released in the closed cavity, the vital adjacent tissues (air sinuses, nerves, vessels) and the burning of bone. Considering, that the root is mechanically open during endodontic treatment the use of micro-suction tips may be found useful in releasing the generated smoke out of the cystic cavity. Diode laser is soft light energy, therefore the expected damage of the surrounding bone should be considered minimal.

Future research need to be carried out to assess the effectiveness of this procedure in clinical trials. Possibly another cost-effective tool may be added to the armamentarium of the dentists for future applications.

## Conclusion

While this technique may have some applications as a possible new technique the research needs to be exhaustive. We are already aware of the capabilities of small fibre laser and the effect of lasers on soft tissues but the effect of lasers used in an enclosed space in living bone is of interest. To be taken further it would presumably need rigorous testing on animals.

## Consent

"Written informed consent was obtained from the patient for publication of this case report and accompanying images. A copy of the written consent is available for review by the Editor-in-Chief of this journal."

## Competing interests

The authors declare that they have no competing interests.

## Authors' contributions

SK analyzed and interpreted the patient data regarding radicular cyst. PK performed the histological examination of the cyst, and was major contributor in performing the laser experiment and writing the manuscript. All authors read and approved the final manuscript.

## References

[B1] Soames JV, Southam JC, Soames JV, Southam JC (1999). Cysts of the jaws and oral soft tissues. Oral Pathology.

[B2] Garcia CC, Sempere FV, Diago MP, Bowen EM (2007). The post-endodontic periapical lesion: histologic and etiopathogenic aspects. Med Oral Patol Oral Cir Bucal.

[B3] Komori T, Yokoyama K, Takato T, Matsumoto K (1997). Clinical application of the erbium: YAG laser for apicoectomy. J Endod.

[B4] Sarver DM, Yanosky M (2005). Principles of cosmetic dentistry in orthodontics: Part 2. Soft tissue laser technology and cosmetic gingival contouring. Am J Orthod Dentofac Orthoped.

[B5] Williams TM, Cobb CM, Rapley JW, Killoy WJ (1995). Histologic evaluation of alveolar bone following CO2 laser removal of connective tissue from periodontal defects. Int J Periodontics Restorative Dent.

